# Atrioventricular Conduction in Mesial Temporal Lobe Seizures

**DOI:** 10.3389/fneur.2021.661391

**Published:** 2021-04-28

**Authors:** Max C. Pensel, Luca M. Basili, Arthur Jordan, Rainer Surges

**Affiliations:** ^1^Department of Psychiatry, University Hospital Bonn, Bonn, Germany; ^2^Department of Neurology and Psychiatry, Sapienza University Rome, Rome, Italy; ^3^Department of Epileptology, University Hospital Bonn, Bonn, Germany

**Keywords:** atrioventricular conduction, mesial temporal lobe epilepsy, cardiac arrhythmia, pre-surgical evaluation, video-EEG monitoring, intracranial depth electrodes

## Abstract

**Purpose:** Asymmetric cerebral representation of autonomic function could help to stratify cardiac complications in people with epilepsy, as some seizures are associated with potentially deleterious arrhythmias including bradycardia and atrioventricular (AV) conduction block. We investigated seizure-related changes in AV conduction and ascertained whether these alterations depend on the hemisphere in mesial temporal lobe epilepsy (mTLE).

**Methods:** EEG and ECG data of people with pharmacoresistant mTLE undergoing pre-surgical video-EEG telemetry with seizures independently arising from both hippocampi, as determined by intracranial depths electrodes were reviewed. RR and PR intervals were measured using one-lead ECG. Statistics were done with paired student's *t*-tests and linear regression analysis. Data are given as mean ± SD.

**Results:** Fifty-six seizures of 14 patients (5 men, age 34.7 ± 9.8 years) were included (2 seizures per hemisphere and patient). There were no differences of absolute PR intervals and HR before and during unilateral ictal activity between left- and right-sided hippocampal seizures. Peri-ictal modulation of AV conduction, however, appeared greater with left-sided seizures, as the slope of the PR/HR correlations was significantly steeper with seizures originating in the left hippocampus. PR lengthening >200 ms or full block did not occur in any seizure.

**Conclusions:** Our data show that on average, PR intervals shortens with mesial temporal lobe seizures with more prominent effects in seizures with left-sided onset, supporting the notion of lateralized cerebral control of cardiac function. The clinical relevance of this subtle finding is unclear but may indicate a lateralized susceptibility to seizure-related AV node dysfunction in mTLE.

## Introduction

Given the intricate overlap of regions involved in the central autonomic nervous system (CAN) and networks that generate or maintain epileptic activity, seizures are frequently accompanied by alterations of cardiac properties and heart activity (1, 2). These changes are mostly benign and may be used to detect and count epileptic seizures using wearable devices (3). Potentially lethal cardiac arrhythmias such as ventricular tachycardia or sustained asystole are rare and contribute to the elevated risk of premature mortality and sudden cardiac death in people with epilepsy (2, 4).

Previous studies have predominantly reported increases and decreases of sinus node activity with subsequent changes in heart rate (HR) as well as lengthening or shortening of cardiac repolarization and QT intervals in association with seizures (4, 5). Whilst ictal bradyarrhythmias and asystole occur rarely and almost exclusively in temporal lobe seizures spreading to the contralateral hemisphere, ictal increases of HR is a frequent phenomenon in temporal and frontal lobe epilepsies of both hemispheres (2). In focal seizures of patients with mesial temporal lobe epilepsy (mTLE), however, QT intervals were lengthened to a greater extent and more often beyond abnormal limits during seizures originating in the left hippocampus, indicating that cerebral control of cardiac repolarization may be asymmetrically represented (6). Little is known about seizure-related alterations of atrioventricular (AV) conduction. In all twelve cases with ictal AV block reported so far, seizures emanated from the left hemisphere (mostly from the temporal lobe), suggesting that delay or inhibition AV conduction is preferentially controlled by CAN networks of the left hemisphere (2, 7). However, direct investigations of changes of PR-Intervals related to seizure activity and hemisphere are lacking so far.

In view of these observations, we hypothesized that seizure-related modulation of AV conduction depends on the side of ictal activity. Such an asymmetric representation of AV node control may serve as lateralizing hint during assessment of seizure-onset zone, but may also help to identify people at an elevated risk for ictal AV block and asystole. In the present study, we investigated seizure-related changes in AV conduction in patients with medically refractory bilateral mTLE who underwent intracranial video-EEG telemetry for pre-surgical diagnostics.

## Materials and Methods

This study is a retrospective audit of EEG and ECG data recorded during standard clinical procedures and was approved as such by the local medical ethics committee (Ethikkommission an der Medizinischen Fakultät der Rheinischen Friedrich-Wilhelms-Universität Bonn, No. 352/12). Patient data of a 12-year period (2000–2011) collected at our tertiary epilepsy center (Department of Epileptology, University Hospital Bonn) were reviewed. All patients suffered from pharmacoresistant mTLE and underwent pre-surgical video-EEG telemetry, with seizures arising from both hippocampi, as assessed by intracranial EEG recordings with depth electrodes. Patients with known cardiac diseases were excluded.

**Figure 1:**
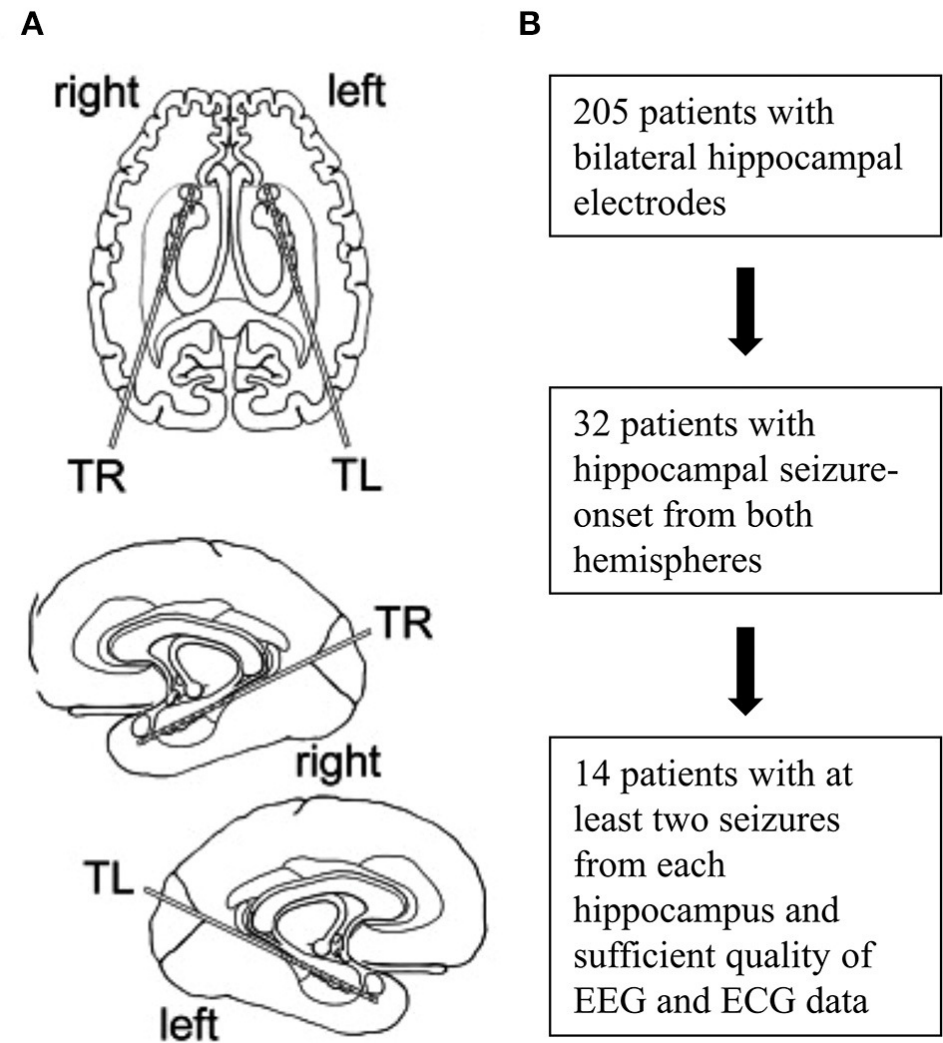
Implantation scheme and flowchart of patient selection. Placement of intracranial depth electrodes for most patients **(A)**. Selection and inclusion of patients **(B)**.

**Figure 2:**
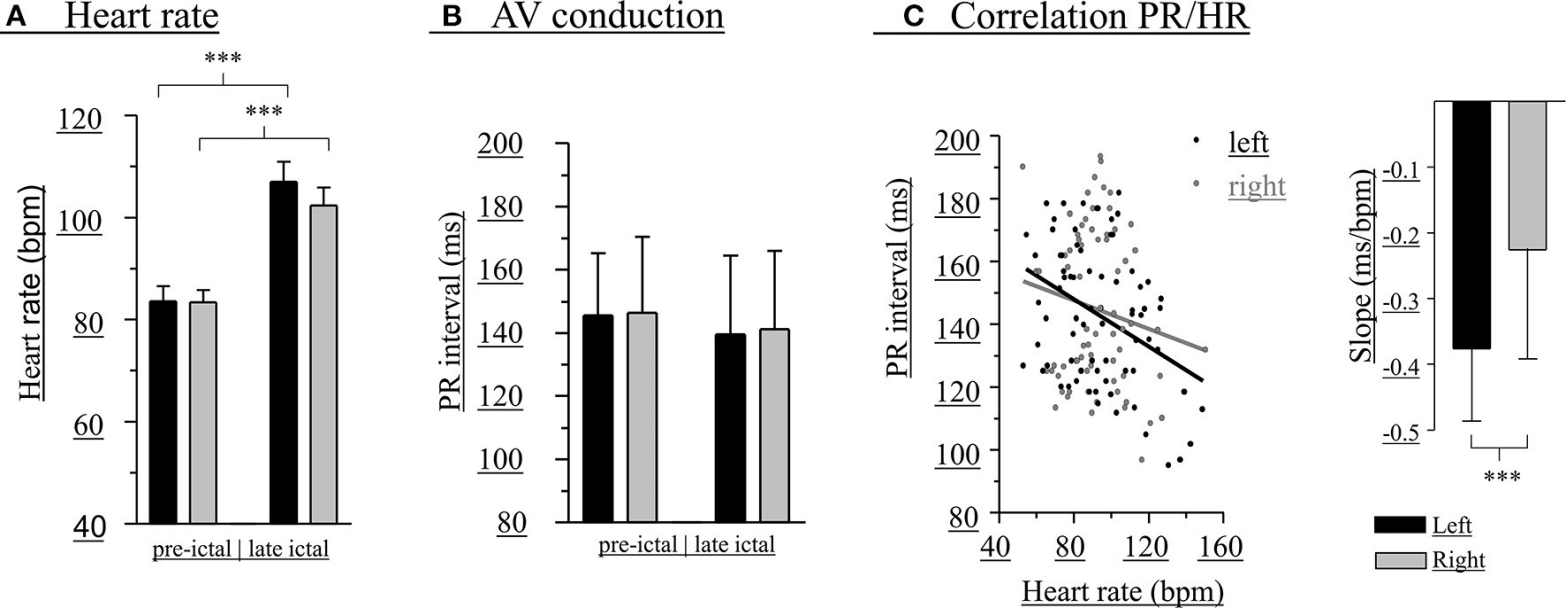
Modulation of PR intervals is greater with left-hippocampal seizures. Heart rate before (left bars) and during (right bars) unilateral hippocampal seizures **(A)**. PR interval before (left bars) and during (right bars) unilateral hippocampal seizures **(B)**. PR interval/HR correlations and slopes (inset) **(C)**. A total of 56 seizures (black bars, left-sided seizures; gray bars, right-sided seizures) of 14 patients was included. ***p < 0.001.

A total of 205 patients with bilateral hippocampal electrodes was eligible. Of these, 32 patients displayed hippocampal seizures with onsets from both hemispheres and 14 patients showed at least two seizures from each hippocampus and had EEG- and ECG-recordings of sufficient quality (especially regarding readability of PR intervals) to be included in the final analysis (Figure 1, Table 1). Two seizures originating from each hippocampus per patient were visually analyzed. If more than two seizures per hemisphere occurred, the two seizures with the greatest HR changes were chosen. All analyzed seizures showed a clear unilateral start. Twelve patients had one 10-contacts hippocampal depth electrode per side stereotactically implanted via a posterior approach (Adtech®, Racine, WI, USA), 1 patient had four 5-contacts depth electrodes implanted from lateral into each hemisphere, covering the amygdala, the hippocampus, the entorhinal cortex and the parahippocampal gyrus. The remaining patient had two 5-contacts electrodes on each side (amygdala and hippocampus), implanted from a lateral approach (Figure 2). Electrode position was confirmed via MRI after implantation. The presurgical assessment included cerebral MRI (1.5 or 3 Tesla), scalp EEG (10–20 system with additional electrodes) and neuropsychological testing, prior to invasive video-EEG telemetry. The EEG data were recorded with a video-synchronized digital system (Stellate Harmonie, Version 5.4, Schwarzer GmbH/Natus, Germany), using up to 128 channels, a 200 Hz sampling rate and a 16-bit digital converter. Band pass filter was applied below 0.016 and above 70 Hz. Seizure onset was defined by the occurrence of ictal EEG pattern. The ECG tracing was recorded simultaneously to the iEEG registration via I-lead ECG, with one electrode placed below each clavicle.

**Table 1:** Clinical features of patients included in this study.

**Patient no**.	**Sex**	**Age^*^a^*^/epilepsy duration^*^b^*^/** **handedness**	**MRI finding**	**Surgery**	**Intracranial electrodes**	**FU^***^c^***^/OC^***^d^***^**
1	M	32/32/R	HS B	No	HC: 1 depth electrode (10 c.) on each side ExHC: None	n.a.
2	M	28/23/L	HS L	SAHE L	HC: 1 depth electrode (10 c.) on each side ExHC: 1 temporo-lateral (16 c.) and 2 temporo-basal (4 c.) strip electrodes on each side	48/I
3	F	24/4/R	HS B	No	HC: 1 depth electrode (10 c.) on each side ExHC: 1 temporo-lateral (4 c.) and 2 temporo-basal (4 c.) strip electrodes on each side	n.a.
4	F	55/33/R	HS B	SAHE R	HC: 1 depth electrode (10 c.) on each side ExHC: 1 temporo-lateral (6 c.) and 2 temporo-basal (4 c.) strip electrodes on each side	53/II
5	M	45/39/L	None	No	HC: 1 depth electrode (10 c.) on each side ExHC: 1 temporo-lateral (4 c.) and 2 temporo-basal (4 c.) strip electrodes on each side	n.a.
6	F	47/7/R	HS B	No	HC: 1 depth electrode (10 c.) on each side ExHipp: None	n.a.
7	F	46/33/R	HS R	SAHE R	HC: 1 depth electrode (10 c.) on each side ExHC: 1 temporo-lateral (6 c.) and 2 temporo-basal (4 c.) strip electrodes on each side	18/II
8	M	22/15/L	None	SAHE L	HC: 1 depth electrode (10 c.) on each side ExHC: 2 temporo-basal (4 c.) strip electrodes on each side, 1 temporo-lateral strip electrode (6 c.) on right side and 1 grid electrode (32 c.) on left side covering Wernicke's area	24/I
9	F	35/30/R	HS B	No	HC: 1 depth electrode (10 c.) on each side ExHC: None	n.a.
10	F	31/30/R	HS B	AHE, TL-resection L	HC: 1 depth electrode (10 c.) on each side ExHC: None	No FU
11	F	34/14/R	HS B	No	HC: 1 depth electrode (10 c.) on each side ExHC: None	n.a.
12	F	28/25/L	HS R	No	HC: 2 depth electrodes (8 c.) on each side ExHC: 2 temporo-basal (4 c.) strip electrodes on each side	n.a.
13	F	31/29/R	HS B	No	HC: 1 depth electrode (10 c.) on each side ExHC: 1 temporo-lateral (6 c.) and 2 temporo-basal (4 c.) strip electrodes on each side	n.a.
14	M	28/15/R	HS L	No	HC: 5 depths electrodes (10 c.) on each side (implanted from lateral). ExHC: 2 frontal strip electrodes (8 c.) on each side	n.a.

a *At telemetry [Y]*,

b *[Y]*,

c *Follow Up [m]*,

d *Outcome [Engel Classification]. B, bilateral; c, electrode contacts; ExHC, extrahippocampal; HC, hippocampal; HS, hippocampal sclerosis; L, left; n.a., not applicable; R, right; SAHE, selective amygdala-hippocampectomy; TL, temporal lobe*.

RR and PR intervals were manually determined at different timepoints (*preictal*: 1 min before EEG seizure-onset, *early ictal*: within the first seconds of seizure activity and *late ictal*: at the end of the unilateral course of the seizure, before spread to the contralateral side or seizure termination). The respective values of RR and PR interval were calculated as a mean of 2–3 successive ECG intervals. After calculating HR values from RR intervals, statistical analyses were performed to compare preictal and ictal data of seizures originating from the left and right hippocampus using paired student's *t*-tests. Furthermore, a bivariate linear regression of HR and PR intervals for right and left-sided seizures was performed. Resulting correlations were considered as a measure of peri-ictal modulation of AV conduction. Analyses and graphs were generated using GraphPad Prism 4 (San Diego, CA 92108, USA). The data underlying the study are available from the corresponding author on reasonable request.

## Results

Recordings of 56 seizures (2 seizures per hemisphere and patient) in 14 patients with refractory mTLE (5 men, age 34.7 ± 9.8 years, epilepsy duration 23.5 ± 10.7 years) were analyzed. Hippocampal sclerosis was found in 12 patients (bilateral in 8 cases), 2 patients had no apparent lesions (Table 1).

Changes of HR were observed during all seizures (Figure 2A). In 64.3% of the seizures HR increases alone occurred, whereas HR increase followed by HR decrease, HR decrease alone, or HR decrease followed by HR increase were observed in 12.5, 12.5, and 10.7%, respectively. Overall, there were statistically significant increases of HR in left (pre-ictal 84 ± 15 bpm, late ictal 107 ± 21 bpm, *t* = −7.34, df = 27, *p* < 0.001) and right-sided (pre-ictal 83 ± 13 bpm, late ictal 102 ± 18 bpm, *t* = −6.48, df = 27, *p* < 0.001) hippocampal seizures.

At the level of individual patients, PR intervals during seizures were consistently shortened (in all 2 seizures per hemisphere and patient) in right-hippocampal seizures of 4 patients and left-hippocampal seizures of 3 patients. Consistent lengthening of PR intervals was found in right-hippocampal seizures of 5 patients and left-hippocampal seizures of 4 patients. Consistent shortening of PR intervals of all seizures of both sides was not observed in any patients, whereas consistent lengthening of PR intervals in all 4 seizures was seen in 1 patient.

At the group level (pooled data), there were no significant differences between left- and right-sided hippocampal seizures regarding absolute PR intervals and HR before (PR intervals: left 146 ± 20 ms, right 146 ± 24 ms, *t* = −0.13, df = 54, *p* = 0.894; HR: left 84 ± 15 bpm, right 83 ± 13 bpm, *t* = 0.07, df = 54, *p* = 0.947) as well as during early (PR intervals: left 143 ± 24 ms, right 146 ± 25 ms, *t* = −0.40, df = 54, *p* = 0.689; HR: left 93 ± 23 bpm, right 92 ± 14 bpm, *t* = 0.31, df = 54, *p* = 0.756) or late (PR intervals: left 138 ± 27 ms, right 141 ± 25 ms, *t* = −0.43, df = 54, *p* = 0.669; HR: left 107 ± 21 bpm, right 102 ± 18 bpm, *t* = 0.90, df = 54, *p* = 0.373) unilateral seizure activity (Figure 2B). Peri-ictal modulation of AV conduction, however, appeared greater during left-sided seizures, as the negative correlation between HR and PR intervals was significantly different than that of right-sided seizures (left:−0.38 ± 0.11 ms/bpm, *r*^2^ = 0.14; right: −0.23 ± 0.17 bpm, *r*^2^ = 0.02; *t* = 6.98, df = 97, *p* < 0.0001; Figure 2C).

## Discussion

In this retrospective clinical study, peri-ictal PR intervals and HR were assessed in patients with pharmacoresistant bilateral mTLE. Importantly, these patients were bilaterally implanted and at least two seizures per side were registered, allowing a comparison between left-and right-hippocampal seizure-related changes of PR intervals, and minimizing the effects of inter- and intra-individual variability. Moreover, the simultaneous iEEG-ECG recording provides optimal ECG resolution regarding time and localization of seizure onsets. While this investigation of seizure and hemisphere related changes of PR-Intervals follows an original approach, it does not come without limitations. Firstly, several other regions involved in the CAN such as the insular cortex, hypothalamus, thalamus and cingulate gyrus were not covered by intracranial EEG electrodes, so that spreading of ictal activity could not be recorded and excluded (8). Secondly, all patients had bilateral mTLE and mostly in association with structurally altered hippocampal tissue, which may modify the contribution of the affected hippocampi to the regulation of autonomic function. Whether our findings apply also to other patient groups remains thus to be elucidated. Thirdly, a systematic electrical stimulation of the implanted electrodes and analysis of ECG alterations was not done, which could have complemented and strengthened our subtle findings.

As expected, all seizures were associated with alterations of HR, mainly chronotropic changes with tachycardia, or mixed patterns, supporting the notion of the hippocampus as an important element within the CAN. Overall, the absolute changes of HR and PR intervals were similar in seizures originating from the left and right hippocampus. Previous studies in apparently healthy subjects revealed an inverse correlation between PR intervals and HR, i.e., PR intervals shorten with increasing HR (9). Intriguingly, when considering the correlation between PR intervals and HR in our patients, PR interval shortening was significantly greater during left-hippocampal seizures, although absolute PR intervals and PR interval changes were within normal limits (4, 9). This subtle finding is unlikely to have a clinical meaning, but may reflect a lateralized representation of the CAN with the left hemisphere being more important in the control of AV conduction. This assumption is also underscored by the finding that AV blocks were exclusively reported with left-sided seizures (although this effect is opposite to the enhanced shortening of PR intervals per HR observed in this study) (2, 7). *In-vivo* experiments in rodents and neuroimaging studies in humans provide structural correlates that link the left mesial temporal lobe to the control of cardiac properties. At the brain stem level, the nucleus ambiguus and raphe nuclei are involved in the modulation of AV conduction (10–12) which, in turn, are connected to structures of the medial temporal lobe (particularly to the subiculum, amygdala, entorhinal cortex, and hippocampus) via the lateral forebrain bundle (1). A meta-analysis of neuroimaging data has highlighted the contribution of the left amygdala to central processing of autonomic function (13), which may explain our finding of a greater impact of left-sided mesial temporal lobe seizures on PR interval shortening.

In conclusion, left-hippocampal seizures appear to be linked to greater changes in cardiac repolarization and AV conduction (6). These alterations are subtle, but underscore the notion of asymmetric lateralization of cortical heart control, which in turn may reflect an elevated risk of disturbed AV conduction in people with left mTLE.

## Data Availability Statement

The data underlying the study are available from the corresponding author on reasonable request.

## Ethics Statement

The studies involving human participants were reviewed and approved by Medinizische Fakultaet Bonn. Written informed consent for participation was not required for this study in accordance with the national legislation and the institutional requirements.

## Author Contributions

LMB and AJ have collected and analyzed data. MP has analyzed the data and drafted the manuscript. RS has designed and supervised the study. All authors have reviewed the draft.

## Conflict of Interest

The authors declare that the research was conducted in the absence of any commercial or financial relationships that could be construed as a potential conflict of interest.
